# The Abnormal Proliferation of Midbrain Dopamine Cells From Human Pluripotent Stem Cells Is Induced by Exposure to the Tumor Microenvironment

**DOI:** 10.1111/cns.70117

**Published:** 2024-11-19

**Authors:** Jun Xue, Dongyan Wu, Yuting Bao, Yifan Wu, Xin Zhang, Liang Chen

**Affiliations:** ^1^ Department of Neurosurgery, Huashan Hospital, MOE Frontiers Center for Brain Science Fudan University Shanghai China; ^2^ National Center for Neurological Disorders, Shanghai Key Laboratory of Brain Function and Restoration and Neural Regeneration, Huashan Hospital Fudan University Shanghai China; ^3^ Institute of Neurology, Huashan Hospital, Shanghai Medical College Fudan University Shanghai China; ^4^ National Clinical Research Center for Aging and Medicine, Huashan Hospital Fudan University Shanghai China

**Keywords:** cell therapies, coculture, midbrain dopamine cells, tumor microenvironment, tumorigenicity tests

## Abstract

**Aims:**

Tumorigenicity is a significant concern in stem cell‐based therapies. However, traditional tumorigenicity tests using animal models often produce inaccurate results. Consequently, a more sensitive method for assessing tumorigenicity is required. This study aimed to enhance sensitivity by exposing functional progenitors derived from human pluripotent stem cells (hPSCs) to the tumor microenvironment (TME) in vitro before transplantation, potentially making them more prone to abnormal proliferation or tumorigenicity.

**Methods:**

Midbrain dopamine (mDA) cells derived from hPSCs were exposed to the TME by coculturing with medulloblastoma. The cellular characteristics of these cocultured mDA cells were evaluated both in vitro and in vivo, and the mechanisms underlying the observed alterations were investigated.

**Results:**

Our findings demonstrated increased proliferation of cocultured mDA cells both in vitro and in vivo. Moreover, these proliferating cells showed a higher expression of Ki67 and SOX1, suggesting abnormal proliferation. The observed abnormal proliferation in cocultured mDA cells was attributed to the hyperactivation of proliferation‐related genes, the JAK/STAT3 pathway, and cytokine stimulation.

**Conclusion:**

This study indicates that exposing functional progenitors to the TME in vitro before transplantation can induce abnormal proliferation, thereby increasing the sensitivity of tumorigenicity tests.

## Graphical abstract

mDA cells exposed to the tumor microenvironment induced abnormal proliferation in vitro and in vivo, which was ascribed to the hyperactivation of proliferation‐related genes, the JAK/STAT3 pathway, and cytokine stimulation. It suggests that exposing functional progenitors to the TME offers a novel strategy to improve the sensitivity of tumorigenicity study.

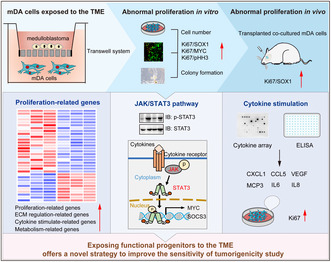

## 1 Introduction

Human pluripotent stem cells (hPSCs) possess the capacity for self‐renewal and differentiation into diverse cell types, making them a promising option for stem cell‐based therapies aimed at treating a wide range of diseases and injuries [1, 2]. However, genetic mutations, residual hPSCs, and abnormal proliferation in cell products can increase the risk of tumor formation, posing a significant safety concern that must be addressed before clinical application [1, 3]. Therefore, conducting sensitive tumorigenicity tests is imperative for stem cell‐based therapies.

Currently, the primary method for assessing tumorigenicity involves the use of animal models [4, 5]. However, traditional in vivo tumorigenicity tests sometimes yield unreliable results. For instance, tumors have been reported in the brain of a boy following fetal neural stem cells (NSCs) transplantation for ataxia telangiectasia [6], in the kidney of a woman after autologous hematopoietic stem cell transplantation for lupus nephritis [7], and in a patient who developed immature teratomas after receiving PSCs‐derived islet beta cells [8]. Given the substantial differences in cell types and gene expression between mice and humans [9, 10], the reliability of conventional animal tests in detecting potential tumorigenicity should be reconsidered. Thus, a novel approach to tumorigenicity tests is required to improve the detection of tumor formation risk.

The tumor microenvironment (TME) is known to play a critical role in the initiation, progression, and metastasis of tumors [11]. Additionally, it has the potential to influence the phenotypic transformation and proliferation of various cell types, such as astrocytes, adipose stem cells, and early NSCs. It has been shown that glioblastoma cells can induce malignant transformation in astrocytes [12], while pediatric glioblastoma cells can inhibit neurogenesis, promote astrogenesis, and induce NSCs migration in cocultures [13]. Moreover, human adipose stem cells have exhibited enhanced proliferation and expression of stemness‐related genes when cocultured with breast cancer cells [14]. However, the impact of the TME on functional progenitors—partially differentiated cells committed to specific cell lineages for therapeutic purposes—remains largely unexplored. We hypothesized that the TME might influence the proliferation of functional progenitors derived from hPSCs. Based on this hypothesis, we proposed exposing functional progenitors to the TME in vitro before transplantation to increase their susceptibility to abnormal proliferation or tumorigenicity.

Stem cell‐based therapy for Parkinson's disease (PD) holds the potential to delay or reverse disease progression by targeting the loss of dopaminergic cells [3, 15, 16]. Clinical trials are currently underway in several countries to explore this promising treatment approach [17, 18, 19, 20, 21, 22, 23]. As previously mentioned, safety is paramount for clinical translation, and strict implementation of tumorigenicity criteria for midbrain dopamine (mDA) cells derived from hPSCs is crucial to prevent graft tumorigenesis. In this study, we selected mDA cells as a representative example of functional progenitors and exposed them to the TME by coculturing with medulloblastoma in a Transwell system. Our research aimed to investigate the impact of the TME on functional progenitors, their potential for tumorigenicity, and the underlying mechanisms.

## 2 Materials and Methods

### 2.1 Maintenance of hPSCs

hPSCs (line H9, passages 30–40) were maintained in NutriStem hPSC XF medium (Biological Industries, 05‐200‐1A, Kibbutz Beit Haemek, Israel) with daily medium changes and cultured on Matrigel (Corning, 354277, Corning, NY)‐coated plates. The cultures were incubated in a humidified environment at 37°C with 5% CO_2_. Upon reaching 70%–80% confluence, H9 cells were digested with EDTA (Invitrogen, 15575‐020, Carlsbad, CA) and passaged onto new Matrigel‐coated plates.

### 2.2 Medulloblastoma Cell Culture

The human medulloblastoma cell line Daoy was generously provided by the Stem Cell Bank, Chinese Academy of Sciences (CAS). Daoy cells were cultured in MEM (Gibco, 11090081, Grand Island, NY) supplemented with 10% fetal bovine serum (FBS, Gibco, 10099141C), nonessential amino acids (NEAA, Gibco, 11140050), GlutaMAX (Gibco, 35050061), and sodium pyruvate (Gibco, 11360070). Upon reaching 70% confluence, Daoy cells were digested with TrypLE (Gibco, 12605028) and passaged onto a new dish.

### 2.3 Generation of mDA Cells

mDA cells were generated from H9 hPSCs following the protocol described by Song et al. [24] with minor modifications (Figure S1A). Briefly, H9 cells were incubated with EDTA for 5 min and dissociated into single cells. Approximately 10,000 cells per spot were seeded onto Matrigel‐coated dishes containing NutriStem medium supplemented with the ROCK inhibitor Y27632 (10 μΜ). On days 1–6, the differentiation medium consisted of DMEM, 15% knockout serum replacement (KSR), and L‐Glutamine (L‐Glu). From days 6 to 12, cells were cultured in DMEM with decreasing concentrations of KSR (11.5% on days 6–8, 7.5% on days 8–10, and 3.75% on days 10–12), along with N2 supplement (0.25% on days 6–8, 0.5% on days 8–10, and 0.75% on days 10–12), L‐Glu, and NEAA. From day 12, the medium was replaced with DMEM/F12 and N2 supplement. β‐Mercaptoethanoer (β‐mer) and SB431542 (10 μΜ) were added from days 1 to 8, and LDN193189 (0.2 μΜ) was included from days 1 to 12. Sonic hedgehog (SHH, 100 ng/mL), FGF8 (100 ng/mL), and purmorphamine (PMN, 2 μΜ) were added from days 2 to 10. CHIR99021 (1 μΜ) was supplemented from days 4 to 14. On day 9, cells were treated with 40 μM quercetin (QC). From day 12, ascorbic acid (AA, 200 μΜ), brain‐derived neurotrophic factor (BDNF, 20 ng/mL), glia‐derived neurotrophic factor (GDNF, 20 ng/mL), dibutyryl cyclic adenosine monophosphate (dbcAMP, 500 μΜ), TGF‐β3 (1 ng/mL), and DAPT (10 μΜ) were included. On day 15 of differentiation, cells were dissociated into single cells with accutase and replated onto poly‐l‐ornithine/fibronectin/laminin‐coated dishes. mDA cells on days 15 and 20 of differentiation were used in this study. The chemicals, peptides, and recombinant proteins used in this study are listed in Table S1.

### 2.4 Transwell Coculture Experiment

**FIGURE 1:**
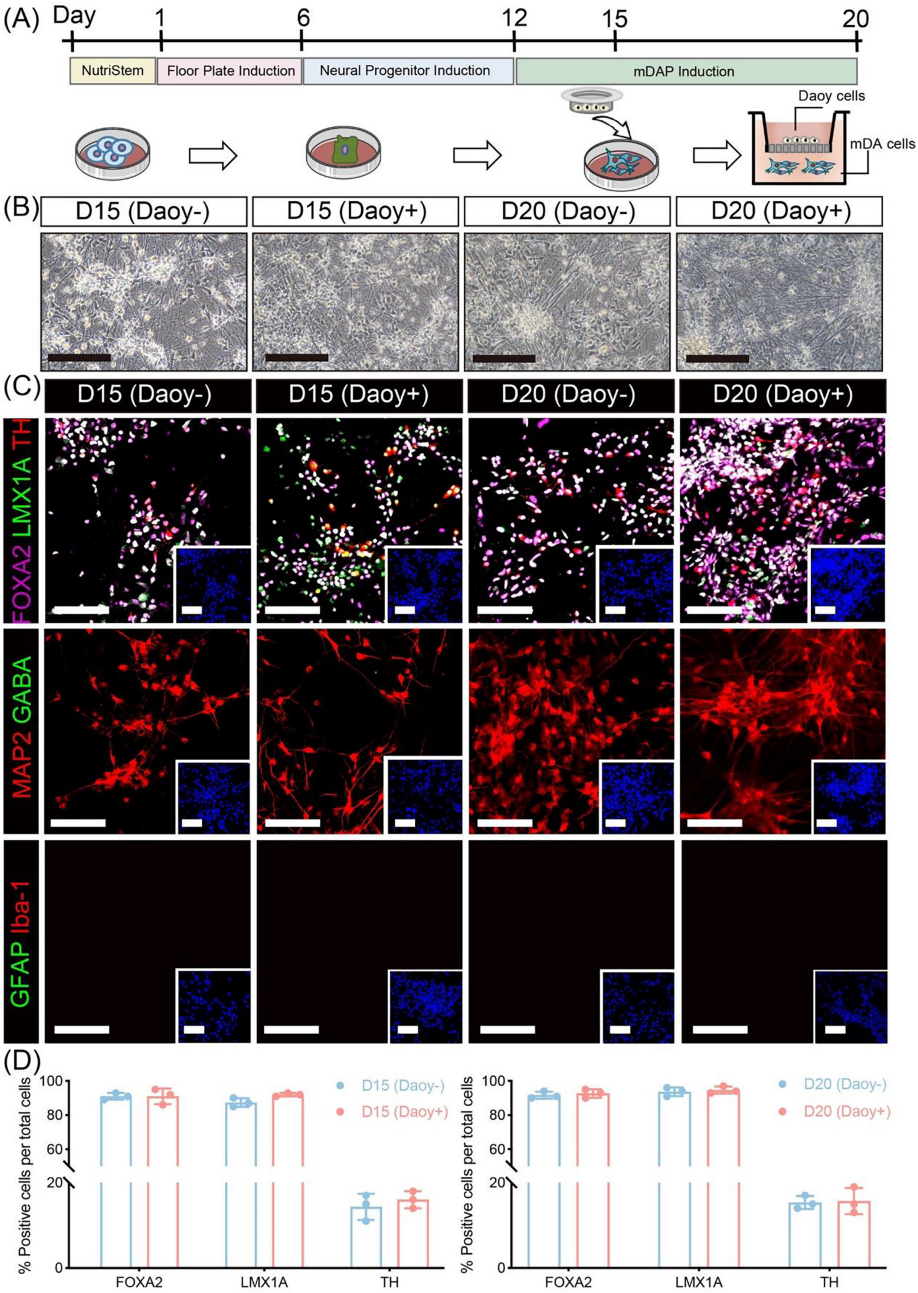
In vitro characterization of mDA cells cocultured with medulloblastoma. (A) Schematic representation of mDA cell differentiation and coculture with medulloblastoma. On days 15 and 20 of differentiation, mDA cells were cocultured with Daoy cells for 4 days in a Transwell system. (B) Bright‐field images of control and cocultured mDA cells beginning from day 15 to day 20 of differentiation. Scale bar: 200 μm. (C) Representative immunofluorescence images of mDAPs (FOXA2/LMX1A), DANs (TH), GABAergic neurons (GABA), mature neurons (MAP2), astrocytes (GFAP), and microglia (Iba‐1) in control and cocultured mDA cells beginning from day 15 to day 20 of differentiation. Scale bar: 80 μm. (D) Quantification of immunofluorescence marker populations in control and cocultured groups. Student's *t*‐test was performed.

We used Transwell systems (Corning, 3450, 3470) with a pore size of 0.4 μm to establish the coculture model, as shown in Figure 1A. mDA cells on days 15 and 20 of differentiation were seeded in the lower compartments of the Transwell plates. Daoy cells were cultured in the insert using a serum‐free medium, with the serum‐free medium alone serving as the control. The Transwell systems were incubated for 4 days without a medium change. After 4 days, we collected the medium for cytokine array, enzyme‐linked immunosorbent assay (ELISA), and mass spectrometry‐based metabolomics. Control and cocultured mDA cells from six‐well Transwell plates (Corning, 3450) were collected for soft agar colony formation assay, nude mice tumor formation assay, stereotactic transplantation, and RNA‐sequencing. Control and cocultured mDA cells collected from 24‐well Transwell plates (Corning, 3470) were used for immunocytochemistry. Each coculture experiment was repeated at least three times.

### 2.5 Immunocytochemistry (ICC)

Cells were fixed with 4% paraformaldehyde (PFA) after being washed with cold PBS and then permeabilized with PBS containing 0.3% Triton X‐100. They were blocked for 1 h in PBS with 0.3% Triton X‐100 and 3% horse serum before adding the primary antibody solutions. After overnight incubation with primary antibodies at 4°C, cells were washed with PBS before adding fluorophore‐conjugated secondary antibodies and Hoechst. Secondary antibodies were incubated at room temperature for 1 h and then washed with PBS. Images were captured using a confocal microscope (Olympus FV3000, Tokyo, Japan). The antibodies and their respective dilutions used in this study are listed in Table S2. Immunofluorescence marker populations were quantified using ImageJ software (National Institutes of Health).

### 2.6 Soft Agar Colony Formation Assay

The soft agar colony formation assay was performed according to the protocol of Du et al. [25]. Briefly, the top and bottom layers of agar were prepared at concentrations of 0.3% and 0.5%, respectively. A total of 5000 control or cocultured mDA cells were reseeded into each well of a 12‐well plate and incubated at 37°C with 5% CO_2_ for 3–4 weeks, with the medium being refreshed every 3 days. Colonies larger than 25 μm in diameter were counted. The experiments were performed in triplicate.

### 2.7 Nude Mice Tumor Formation Assay

All animal experiments were performed according to the guidelines for the Care and Use of Laboratory Animals of Fudan University School of Medicine and were approved by the Institutional Animal Care and Use Committee (202009007S). For the tumor formation assay, 6‐ to 8‐week‐old male Balb/c nude mice (Charles River) were used, as described previously [17]. The mice were randomly divided into five groups without considering any other variables: injected with control mDA cells beginning from day 15 to day 20 of differentiation, injected with cocultured mDA cells beginning from day 15 to day 20 of differentiation, and injected with positive control H9 cells. The cells were dissociated into single cells and subcutaneously injected into the back at a concentration of 200,000 cells/mouse. The mice were monitored every other day for tumor nodule formation and were sacrificed 8 weeks postinjection. Each group consisted of at least five mice.

### 2.8 Stereotactic Transplantation

Additionally, 8‐ to 10‐week‐old male NOD SCID mice (NOD. CB17‐Prkdcscid/NCrCrl, Charles River) were used for stereotactic transplantation, as described previously [24]. The mice were randomly divided into four groups without considering any other variables: transplanted with control mDA cells beginning from day 15 to day 20 of differentiation, and transplanted with cocultured mDA cells beginning from day 15 to day 20 of differentiation. The mice were anesthetized with isoflurane delivered at a flow rate of 1.0 L/min, and anesthesia depth was monitored and adjusted throughout the procedure to maintain a consistent level of anesthesia. A total of 4 μL (50,000 cells/μL) of control or cocultured mDA cells were injected into the bilateral striatum (AP: 0.5 mm, ML: ± 1.8 mm, DV: −3.2 mm) of NOD SCID mice. The needle was kept in the target for 5 min and withdrawn over 5 min. After surgery, the mice were monitored on a warm pad until recovery, and 0.9% sodium chloride was administrated intraperitoneally for 3 days. Eight weeks post‐transplantation, the NOD SCID mice were sacrificed for graft analysis. The experiments were conducted with at least five mice in each group.

### 2.9 Brain Sectioning, Hematoxylin and Eosin (H&E) Staining, Immunofluorescence (IF)

Mice were sacrificed by perfusion with cold PBS followed by cold 4% PFA. The brains were removed and post fixed for an additional 2 h, then dehydrated and embedded in paraffin wax. The paraffin blocks were sectioned at a thickness of 4 μm to collect coronal striatum slices. The slices were dewaxed and dehydrated before H&E and IF staining. H&E staining was performed according to the manufacturer's instructions (Yeasen, 60524ES60, Shanghai, China) and visualized using a research slide scanner (Olympus VS200). For brain section IF, slices underwent additional antigen retrieval procedures. Subsequently, the slices were incubated with 0.3% PBST three times and blocked in 3% horse serum for 1 h. Sections were incubated with primary antibodies at 4°C overnight and then further incubated with secondary antibodies after washing off the primary antibodies. Images of brain sections were captured using a confocal microscope (Olympus FV3000). The antibodies and their respective dilutions used in this study are listed in Table S2. Immunofluorescence marker populations were quantified using ImageJ software (National Institutes of Health).

### 2.10 RNA‐Sequencing

Total RNA from control and cocultured mDA cells beginning from day 15 to day 20 of differentiation (*n* = 3) was extracted using Trizol reagent. A total of 2 μg RNA per sample was used as input material for RNA sample preparations. RNA‐sequencing was performed by Annoroad Gene Technology Corporation. The read count for each gene in each sample was calculated using HTSeq v0.6.0, and Fragments Per Kilobase per Million (FPKM) were computed to estimate the gene expression levels of genes in each sample. DEGseq was used for differential gene expression analysis between two samples without biological replicates, while DESeq2 was applied for analysis between samples with biological replicates, based on the assumption of a negative binomial distribution for the count values. Genes with *q* ≤ 0.05 and |log_2_ ratio| ≥ 1 were identified as differentially expressed genes (DEGs). Gene Ontology (GO) enrichment of DEGs was conducted using the hypergeometric test, with the *p*‐value adjusted to *q*‐value and the data background comprising genes from the whole genome. GO terms with *q* < 0.05 were considered significantly enriched. Kyoto Encyclopedia of Genes and Genomes (KEGG) enrichment of DEGs was also conducted using the hypergeometric test, with *p*‐values adjusted by multiple comparisons to *q*‐values. KEGG terms with *q* < 0.05 were considered significantly enriched. The raw data have been submitted to the Sequence Read Archive (SRA) database of NCBI (PRJNA1066752).

### 2.11 Immunoblotting

Cells were lysed in NP‐40 buffer containing protease inhibitors (Selleck, B14001, Shanghai, China) and phosphatase inhibitors (Selleck, B15001). Samples were prepared with loading buffer, separated by electrophoresis on a 10% SDS‐PAGE gel, and transferred onto PVDF membranes. The membranes were blocked with 5% BSA in 0.1% TBST for 1 h at room temperature, followed by incubation with primary antibodies overnight at 4°C. The membranes were then washed with 0.1% TBST and incubated with secondary antibodies for 1 h. After further washing with 0.1% TBST, the blots were visualized using the enhanced chemiluminescence method. All experiments were performed in triplicate. The antibodies and their respective dilutions used in this study are listed in Table S2.

### 2.12 Mass Spectrometry‐Based Metabolomics

The mass spectrometry‐based metabolomics procedure was performed according to Yuan et al. [26]. Briefly, 1 mL of methanol:acetonitrile:water (2:2:1) solution was added to the collected cells (100,000 cells per sample). The cells were lysed using liquid nitrogen three times and maintained at −20°C for 1 h. The samples were then centrifuged at 15,000 *g* for 15 min at 4°C. The supernatant was collected and freeze‐dried under vacuum. Subsequently, 100 μL of acetonitrile were added to re‐dissolve the sample, followed by vertexing for 30 s and sonication for 5 min. The supernatant was transferred to an injection vial and analyzed after centrifugation at 15,000 *g* for 15 min. The samples were analyzed using LC–MS (Thermo Scientific Q Exactive, Waltham, MA) at the CAS Center for Excellence in Molecular Plant Science.

### 2.13 Cytokine Array

A human cytokine antibody array membrane (Abcam, ab133998, Cambridge, MA) was used to determine cytokine secretion levels in different culture media. Culture media from Daoy cells, mDA cells, and cocultured mDA cells were collected and centrifuged at 300 *g* for 5 min at 4°C. The supernatant was then centrifuged at 2000 *g* for 20 min at 4°C. The membranes were blocked with the provided buffer for 1 h and subsequently incubated with 1 mL of culture medium at 4°C overnight. After washing, the membranes were incubated with the biotin‐conjugated anti‐cytokine overnight at 4°C. Following further washing, the membranes were incubated with horseradish peroxidase‐conjugated streptavidin for 2 h at room temperature. Chemiluminescence was detected using the gel documentation system (Gel Doc XR+ System; Bio‐Rad, Hercules, CA). Relative cytokine intensities were quantified using ImageJ software (National Institutes of Health). The relative intensities of cytokines in cocultured mDA cells were compared with those in mDA cells alone. All experiments were conducted in triplicate.

### 2.14 Enzyme Linked Immunosorbent Assay (ELISA)

The concentration of cytokines in the culture supernatant was determined using ELISA, following the manufacturer's instructions. Absorbance was measured at 450 nm using a microplate reader (Tecan Infinite 200 PRO, Switzerland), with 540 nm set as the calibration wavelength. Each experiment was performed in triplicate. The ELISA kits used in this study are listed in Table S3.

### 2.15 Treatment of JAK/STAT3 Inhibitor

To block the JAK/STAT3 signaling pathway, 20 μM Ruxolitinib was applied to mDA cells 1 h prior to coculture with medulloblastoma cells. The treatment was refreshed every other day for a total of 4 days. Each experiment was conducted at least three times.

### 2.16 Cytokine Treatment to the mDA Cells

On day 15 or day 20 of differentiation, mDA cells were treated with varying concentrations of cytokines (80, 160, 320 ng/mL). The cytokine treatment was refreshed every other day for a total of 4 days. The proliferation of mDA cells exposed to cytokines was analyzed by ICC, with each experiment conducted in triplicate.

### 2.17 Statistical Analysis

GraphPad Prism 10 (GraphPad Software, USA) was used for data analysis. A 95% confidence interval was applied, and results were presented as mean ± standard deviation (Mean ± SD). The Shapiro–Wilk test was used to assess normality. A *t*‐test was used for comparisons between two samples, and one‐way Analysis of Variance (ANOVA) was employed for comparisons among multiple samples. *p*‐values < 0.05 were considered statistically significant, with significance levels set as *p < 0.05, **p < 0.01, and ***p < 0.001.

## 3 Results

### 3.1 Abnormal Proliferation of Cocultured mDA Cells Was Induced In Vitro

mDA cells derived from hPSCs were generated following the protocol established by Kwang‐Soo Kim's group with minor modifications (Figure S1A,B) [24]. The mDA cells used for PD cell therapy consisted primarily of midbrain dopamine progenitors (mDAPs) along with a smaller population of mature dopamine neurons (DANs). As shown in Figure S1C,D, approximately 60% of the total cells expressed MAP2, 15% expressed the DANs marker TH, and over 90% expressed the mDAPs markers EN1, FOXA2, and LMX1A, indicating successful generation of mDA cells.

The basic characterization of cocultured mDA cells was confirmed. As shown in Figure 1B, no significant morphological differences were observed in the mDA cells after coculture with medulloblastoma for 4 days. Additionally, the proportions of both mDAPs and DANs remained stable in cocultured mDA cells, with no significant changes observed beginning from day 15 to day 20 of differentiation (Figure 1C,D). Furthermore, the cocultured mDA cells did not differentiate into GABAergic neurons (GABA^+^), astrocytes (GFAP^+^), or microglia (Iba‐1^+^) as shown in Figure 1C.

**FIGURE 2:**
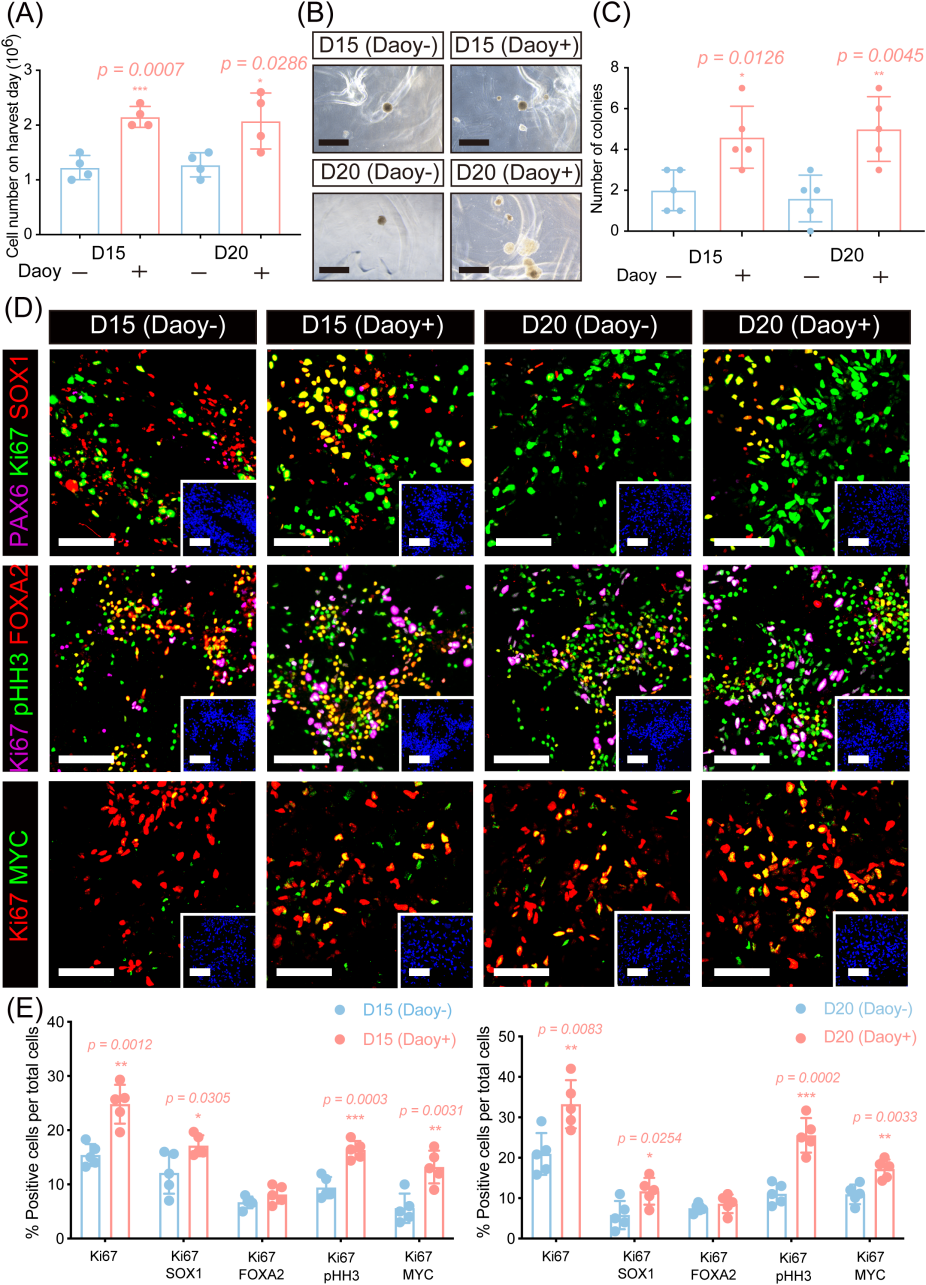
Abnormal proliferation of cocultured mDA cells was induced in vitro. (A) Quantification of cell number on harvest day in control and cocultured groups. (B) Representative bright‐field images from the in vitro soft agar colony formation assay. Scale bar: 800 μm. (C) Number of colonies in the control and cocultured groups beginning from day 15 a day 20 of differentiation. (D) Representative immunofluorescence images of neural precursors (SOX1), cell proliferation markers (Ki67/pHH3/MYC), and dorsal patterning marker (PAX6) in the control and cocultured mDA cells beginning from day 15 to day 20 of differentiation. (E) Quantification of immunofluorescence marker populations in control and cocultured groups. Scale bar: 80 μm. *p < 0.05, **p < 0.01, ***p < 0.001. Student's *t*‐test was performed.

Stem cells have the ability to proliferate in culture for extended periods; however, excessive or abnormal proliferation poses risks and contributes to tumorigenicity. Thus, the proliferation of cell products in vitro and in vivo is a crucial factor in tumorigenicity tests for stem cell‐based therapies [17, 18, 19, 23, 24]. As shown in Figure 2A, the number of mDA cells significantly increased on the harvested day in the cocultured group (D15 control vs. D15 cocultured, 1.23 **×** 10^6^ ± 0.22 vs. 2.15 **×** 10^6^ ± 0.19, *p* = 0.0007; D20 control vs. D20 cocultured, 1.28 **×** 10^6^ ± 0.22 vs. 2.01 **×** 10^6^ ± 0.51, *p* = 0.0286). Additionally, the percentage of Ki67‐positive mDA cells significantly increased in the cocultured groups both beginning from day 15 to day 20 of differentiation (D15 control vs. D15 cocultured, 15.43% ± 2.03% vs. 24.79% ± 3.59%, *p =* 0.0012; D20 control vs. D20 cocultured, 20.94% ± 5.16% vs. 33.22% ± 5.96%, *p* = 0.0083, Figure 2D,E). Moreover, the percentage of Ki67/phosphohistone3 (pHH3^+^) co‐positive cells was significantly higher in the cocultured group (D15 control vs. D15 cocultured, 9.41% ± 1.97% vs. 16.33% ± 1.69%, *p* = 0.0003; D20 control vs. D20 cocultured, 11.08% ± 2.57% vs. 25.54% ± 4.29%, *p* = 0.0002, Figure 2D,E), indicating an increase in actively dividing mDA cells in the cocultured group, as pHH3 is exclusively expressed in mitotic cells in the G2/M phase [27]. Furthermore, the expression of MYC, a known oncogene involved in cell cycle regulation, proliferation, and survival [28, 29], was also examined. Beginning from day 15 of differentiation, the percentage of Ki67/MYC co‐positive cells was 13.20% ± 3.03% in the cocultured group, compared to 5.60% ± 2.70% in the control group. Similarly, beginning from day 20 of differentiation, 17.18% ± 2.39% of mDA cells in the cocultured group co‐expressed Ki67/MYC, while 10.94% ± 2.40% co‐expressed Ki67/MYC in the control group (D15 control vs. D15 cocultured, *p* = 0.0031; D20 control vs. D20 cocultured, *p* = 0.0033). These findings revealed that the cell proliferation of mDA cells was enhanced after coculture with medulloblastoma.

We then performed an in‐depth analysis of the proliferating mDA cells to determine whether their increased proliferation upon coculturing resulted in enhanced differentiation or abnormal proliferation. As shown in Figure 1C and Figure 2D,E, the expression of mDAPs (FOXA2^+^, LMX1A^+^), DANs (TH^+^), and proliferating mDAPs (Ki67^+^/FOXA2^+^) showed no significant differences between the control and cocultured groups, suggesting that the differentiation capability of mDA cells was not enhanced following coculture with medulloblastoma. In contrast, the percentage of neural precursor marker SOX1 costained with Ki67 significantly increased in cocultured mDA cells compared with the control group (D15 control vs. D15 cocultured, 12.11% ± 3.83% vs. 17.14% ± 1.93%, *p* = 0.0305; D20 control vs. D20 cocultured, 5.80% ± 3.50% vs. 11.70% ± 3.31%, *p* = 0.0254). These findings suggest that the enhanced proliferation of mDA cells involved additional early neural precursor cells.

Additionally, in the soft agar colony formation assay, cocultured mDA cells formed an average of 4.60 ± 1.52 and 5.00 ± 1.58 colonies beginning from day 15 to day 20 of differentiation, respectively, while the control group formed an average of 2.00 ± 1.00 and 1.60 ± 1.14 colonies (Figure 2B,C, D15 control vs. D15 cocultured, *p* = 0.0126; D20 control vs. D20 cocultured, *p* = 0.0045). Soft agar colony formation assays are widely used to assess cell differentiation, transformation, and tumorigenesis by evaluating the anchorage‐independent growth capacity of cells to detect their tumorigenic potential [25]. Therefore, these results indicate that the abnormal proliferation and potential tumorigenicity features were acquired by mDA cells after coculture with medulloblastoma.

In summary, in vitro analysis showed a significant increase in cell proliferation in cocultured mDA cells. These proliferating cells were predominantly early neural precursor cells with enhanced colony formation ability, indicating abnormal proliferation and potential tumorigenic characteristics, posing a risk for tumor formation in cocultured mDA cells.

### 3.2 Abnormal Proliferation Presented After Transplantation of Cocultured mDA Cells

To further evaluate the proliferation and tumorigenic potential of cocultured mDA cells in vivo, we conducted assays involving subcutaneous injection of both control and cocultured mDA cells into Balb/c nude mice, as well as striatal transplantation into NOD SCID mice.

In the nude mice tumor formation assay, only the positive control H9 cells formed teratomas with three germ layers after subcutaneous injection (Figure S2). No apparent tumor formation was observed in Balb/c nude mice injected with either mDA cells or cocultured mDA cells after 8 weeks.

**FIGURE 3:**
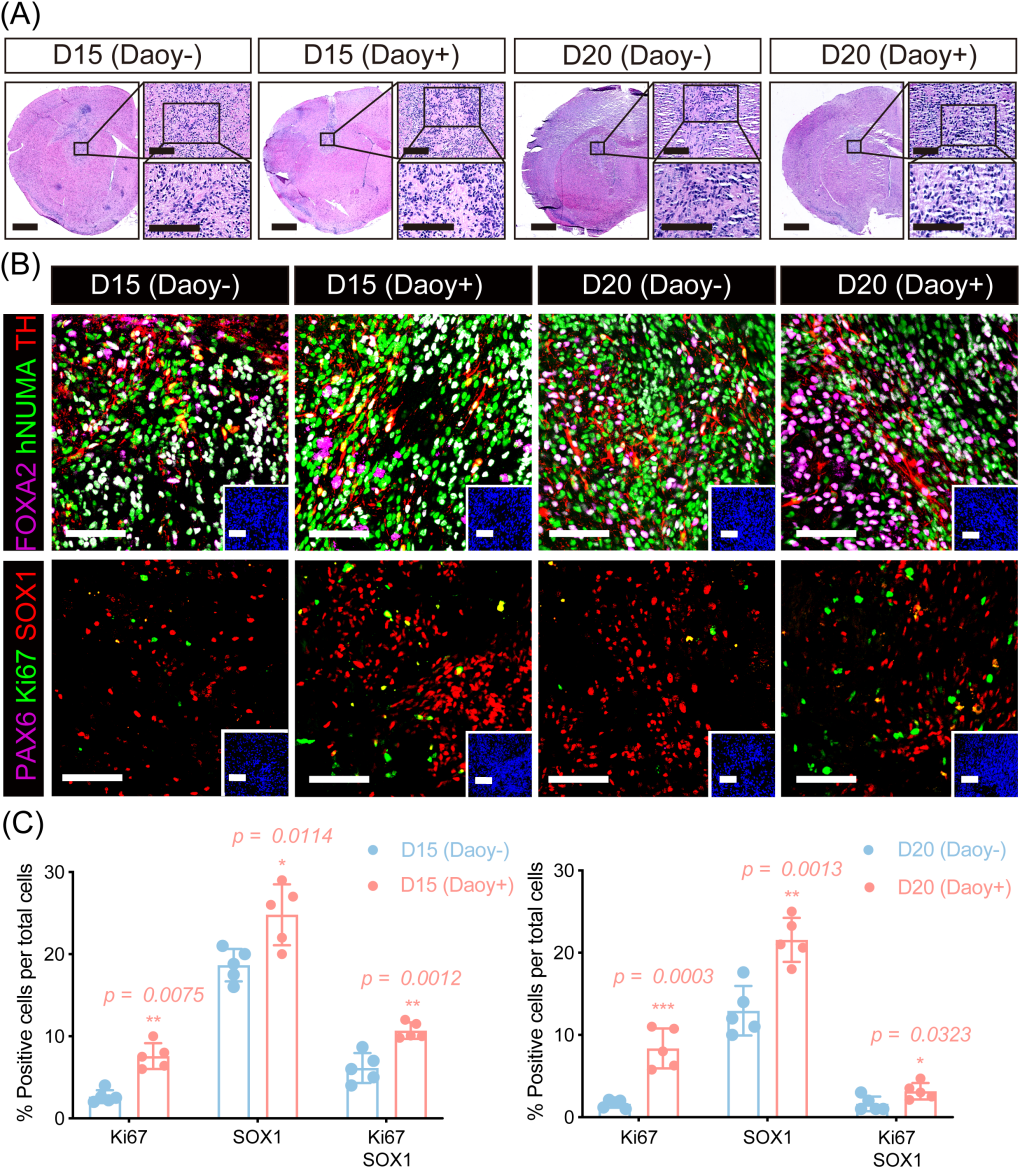
Abnormal proliferation presented after transplantation of cocultured mDA cells. (A) H&E staining of NOD SCID mouse brains after transplantation of control and cocultured mDA cells beginning from day 15 to day 20 of differentiation. Scale bar: 500 μm. (B) Immunofluorescence images showing TH, FOXA2, Ki67, SOX1, PAX6, and hNUMA in grafted brain slices. Scale bar: 80 μm. (C) Quantification of immunofluorescence marker populations in control and cocultured grafted brain slices. *p < 0.05, **p < 0.01, ***p < 0.001. Student's *t*‐test was performed.

In the bilateral striatum transplantation of control and cocultured mDA cells, TH^+^ cells in the graft were colabeled with human‐specific nuclear mitotic apparatus protein (hNUMA) and FOXA2. There was no evidence of tumor formation in either group (Figure 3A). The percentage of FOXA2 and TH‐positive cells did not significantly differ between grafts derived from control and cocultured mDA cells (Figure 3B,C). Additionally, staining for Ki67, SOX1, and PAX6 was performed to assess the proliferation of grafted cells. As shown in Figure 3B,C, the percentage of Ki67‐positive cells in the control group, beginning from day 15 to day 20 of differentiation, was 2.66% ± 0.79% and 1.62% ± 0.49%, respectively. In contrast, the percentage of Ki67‐positive cells significantly increased in the cocultured group both beginning from day 15 to day 20 of differentiation (D15 cocultured 7.58% ± 1.58%, *p* = 0.0075; D20 cocultured 8.37% ± 2.43%, *p* = 0.0003). Furthermore, the percentage of SOX1‐positive cells and Ki67/SOX1 double‐positive cells was significantly higher in the grafts derived from cocultured mDA cells both beginning from day 15 to day 20 of differentiation (D15 control vs. D15 cocultured: SOX1, 18.66% ± 1.98% vs. 24.80% ± 3.70%, *p* = 0.0114; Ki67/SOX1, 6.14% ± 1.82% vs. 10.68% ± 1.00%, *p* = 0.0012; D20 control vs. D20 cocultured: SOX1, 12.92% ± 3.01% vs. 21.57% ± 2.67%, *p* = 0.0013; Ki67/SOX1, 1.60% ± 0.89% vs. 3.16% ± 1.01%, *p* = 0.0323).

Overall, the cocultured mDA cells exhibited a sustained increase in proliferation with early neural precursor cells in vivo, suggesting the presence of abnormal proliferation in the grafts. However, this increased abnormal proliferation was not sufficient to induce tumor formation.

### 3.3 Proliferation‐Related Genes and JAK/STAT3 Pathway Were Hyperactivated in Cocultured mDA Cells

RNA was extracted from the cocultured and control groups beginning from day 15 to day 20 of differentiation, respectively. Subsequently, RNA‐sequencing was conducted to identify the molecular components that may be responsible for the abnormal proliferation of cocultured mDA cells.

**FIGURE 4:**
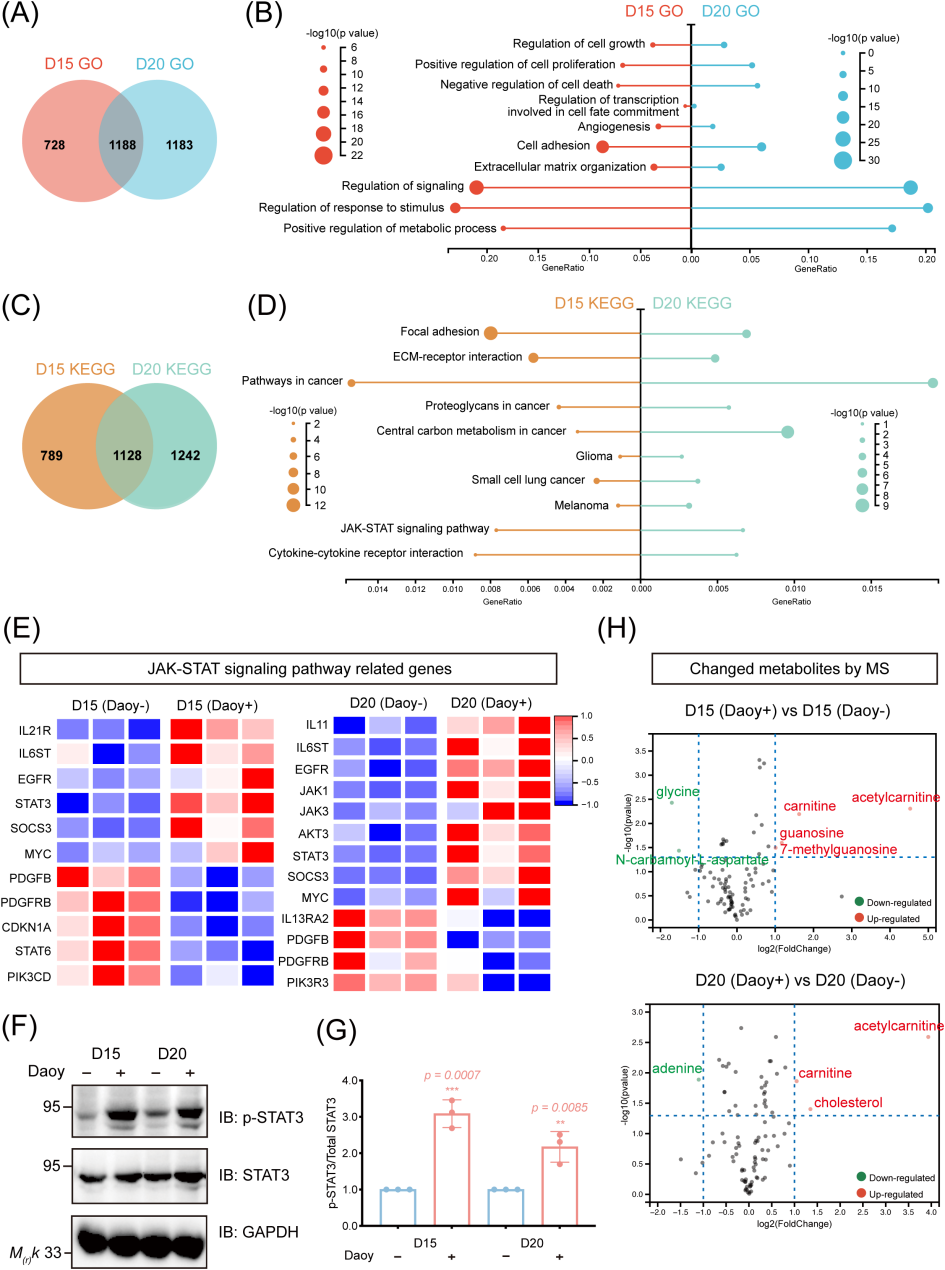
Proliferation‐related genes and the JAK/STAT3 pathway were hyperactivated in cocultured mDA cells. (A) Venn diagram of GO terms enriched in cocultured mDA cells beginning from day 15 to day 20 of differentiation. (B) Selected GO terms enriched in cocultured mDA cells beginning from both day 15 to day 20 of differentiation. (C) Venn diagram of KEGG terms enriched in cocultured mDA cells beginning from day 15 to day 20 of differentiation. (D) Selected KEGG terms enriched in cocultured mDA cells beginning from both day 15 to day 20 of differentiation. (E) Heatmap of significantly altered genes involved in the JAK/STAT signaling pathway. (F) Representative immunoblotting images of JAK/STAT3 signaling pathway proteins in control and cocultured groups. (G) Statistical analysis of immunoblotting data showing the ratio of phosphorylated protein to total protein expressed as fold changes over the basal level. **p < 0.01, ***p < 0.001. Student's *t*‐test was performed. (H) Volcano plot illustrating variance in metabolites between cocultured and control groups in terms of fold change and significance.

As illustrated in the volcano plot (Figure S3), 192 genes were significantly up‐regulated, and 119 genes were significantly down‐regulated in cocultured mDA cells beginning from day 15 of differentiation, whereas 470 genes were up‐regulated and 208 were down‐regulated in cocultured mDA cells beginning from day 20 of differentiation. Moreover, Gene Ontology (GO) analysis showed that a total of 1188 identical GO terms were enriched in cocultured groups, beginning from day 15 to day 20 of differentiation, as shown in Figure 4A. Similarly, Kyoto Encyclopedia of Genes and Genomes (KEGG) analysis indicated that 1128 identical KEGG terms were present in the cocultured groups (Figure 4C).

RNA‐sequencing analysis further confirmed that cocultured mDA cells exhibited abnormal cell proliferation. As illustrated in Figure 4B, GO terms such as “Regulation of cell growth,” “Positive regulation of cell proliferation,” and “Negative regulation of cell death” were enriched, indicating enhanced cell proliferation in cocultured mDA cells. Additionally, GO terms such as “Angiogenesis” and “Cell adhesion,” along with KEGG terms (Figure 4D) such as “Focal adhesion,” “Pathways in cancer,” “Proteoglycans in cancer,” “Central carbon metabolism in cancer,” “Glioma,” “Small cell lung cancer,” and “Melanoma,” were enriched in the cocultured mDA cells, suggesting that these cells exhibited abnormal proliferation with tumor‐like properties.

KEGG analysis (Figure 4D) revealed the involvement of the Janus kinase/signal transducer and activator of transcription three (JAK/STAT3) pathway in the cocultured groups. Subsequently, a heatmap based on the expression of genes associated with the JAK/STAT3 pathway was generated. As shown in Figure 4E, the stimulator of this signaling pathway, IL6ST, was highly expressed in the cocultured mDA cells. Additionally, the signal‐transmitting tyrosine kinases JAK1 and JAK3, along with the transcription factor STAT3, were elevated in the cocultured group. Downstream signaling molecules, including MYC and SOCS3, were also increased in the cocultured mDA cells. Furthermore, immunoblotting was performed to confirm the activation of the JAK–STAT3 pathway. As shown in Figure 4F,G, the ratio of phosphorylated to total STAT3 was significantly higher in the cocultured groups compared to the control groups (D15 control vs. D15 cocultured, 1.00 ± 0.00 vs. 3.09 ± 0.38, *p* = 0.0007; D20 control vs. D20 cocultured, 1.00 ± 0.00 vs. 2.17 ± 0.42, *p* = 0.0085), indicating hyperactivation of the JAK/STAT3 pathway in cocultured mDA cells. Additionally, cocultured mDA cells were treated with 20 μM Ruxolitinib to inhibit the JAK/STAT3 pathway and elucidate its role in enhancing cell proliferation. As shown in Figure S4, suppression of the JAK/STAT3 pathway by Ruxolitinib significantly reduced the proliferation of cocultured mDA cells in vitro (D15 cocultured vs. D15 cocultured + Ruxolitinib, 27.77% ± 0.04% vs. 13.16% ± 0.04%, *p* = 0.0116; D20 cocultured vs. D20 cocultured + Ruxolitinib, 31.98% ± 0.02% vs. 17.58% ± 0.06%, *p* = 0.0138). Overall, considering the elevated MYC expression in cocultured groups (Figure 2D), we concluded that hyperactivation of the JAK/STAT3/MYC pathway plays a critical role in the enhanced proliferation of cocultured mDA cells.

As the GO term “Positive regulation of metabolic process” (Figure 4B) was enriched in our RNA‐sequencing data and previous studies have highlighted the critical roles of glycolysis [30] and lipid metabolism [31] in cell proliferation and tumor progression, we hypothesized that tumor‐related metabolites might contribute to the abnormal proliferation of cocultured mDA cells. Therefore, we conducted a targeted metabolomics analysis focused on tumor‐related metabolites. As shown in Figure 4H, acetylcarnitine and carnitine were notably up‐regulated in the cocultured mDA cells. Carnitine serves as an essential cofactor in the mitochondrial oxidation of fatty acids, which generates cellular energy [32], while acetylcarnitine plays a major role in fatty acid oxidation and energy metabolism [33]. We propose that the increased acetylcarnitine/carnitine ratio may contribute to the enhanced proliferation of cocultured mDA cells, potentially through the enhancement of energy metabolism pathways.

In summary, we suggest that the hyperactivation of the JAK/STAT3/MYC pathway and the increased expression of acetylcarnitine/carnitine may contribute to the abnormal proliferation of cocultured mDA cells.

### 3.4 Cytokines Were Detected in the Medium of Cocultured mDA Cells and Enhanced the Proliferation of mDA Cells

RNA‐sequencing analysis indicated that cocultured mDA cells were associated with intercellular interactions, as evidenced by the enrichment of the GO term “Extracellular matrix organization” (Figure 4B) and the KEGG term “ECM‐receptor interaction” (Figure 4D). These results suggested that the extracellular microenvironment, specifically the TME in this study, may significantly impact the abnormal proliferation of cocultured mDA cells. Furthermore, the enrichment of the GO term “Regulation of response to stimulus” and the KEGG term “Cytokine‐cytokine receptor interaction” suggested that the cocultured mDA cells were exposed to substantial external stimuli, likely cytokines secreted by medulloblastoma cells. Cytokines play a crucial role in cell communication within the TME, and their abnormal generation is a common downstream consequence of oncogenic changes in both malignant and nonmalignant cells [34]. We propose that cytokines secreted by medulloblastoma cells may also contribute to the abnormal proliferation of mDA cells.

**FIGURE 5:**
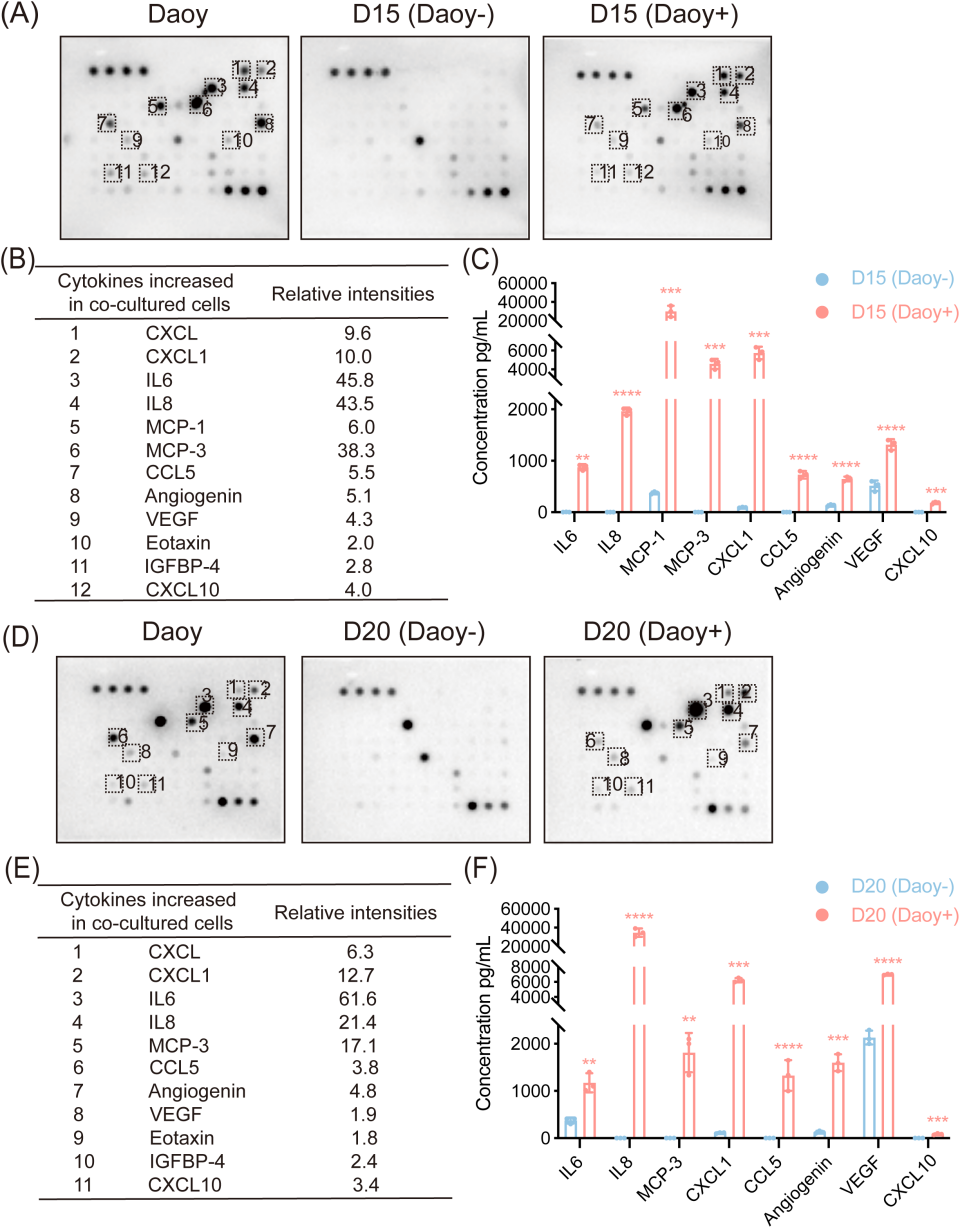
Cytokines were detected in the medium of cocultured mDA cells. Human cytokine antibody array for the culture supernatant of Daoy, mDA cells, and cocultured mDA cells beginning from day 15 of differentiation (A) and day 20 of differentiation (D). Relative intensities of cytokines increased in cocultured mDA cells compared with mDA cells alone from day 15 of differentiation (B) and day 20 of differentiation (E). The concentration of upregulated cytokines in the cocultured mDA cells and mDA cells alone at different time points by ELISA analysis (C&F). For (C) and (F), **p < 0.01, ***p < 0.001, ****p < 0.0001. Student's *t*‐test was performed.

To verify this hypothesis, we employed a human cytokine antibody array to screen for the presence of cytokines in the medium of cocultured mDA cells. As shown in Figure 5A,B, Interleukin 6 (IL6), IL8, Monocyte Chemotactic Protein‐3 (MCP‐3), C‐X‐C Motif Chemokine 1 (CXCL1), MCP‐1, C‐C Motif Chemokine Ligand 5 (CCL5), Angiogenin, VEGF, CXCL10, Insulin‐like Growth Factor Binding Protein‐4 (IGFBP‐4), and Eotaxin were highly expressed in the cocultured mDA cells beginning from day 15 of differentiation and in the Daoy cells compared to the mDA cells alone. ELISA analysis confirmed a significant up‐regulation of these cytokines in the medium from cocultured mDA cells (Figure 5C). Similarly, cytokines IL6, IL8, MCP‐3, CXCL1, Angiogenin, CCL5, CXCL10, IGFBP‐4, VEGF, and Eotaxin showed a notable increase in the cocultured mDA cells beginning from day 20 of differentiation (Figure 5D,E), and these results were further validated by ELISA (Figure 5F). Collectively, these findings revealed that the medium of cocultured mDA cells led to the expression of multiple cytokines, likely secreted from the medulloblastoma.

**FIGURE 6:**
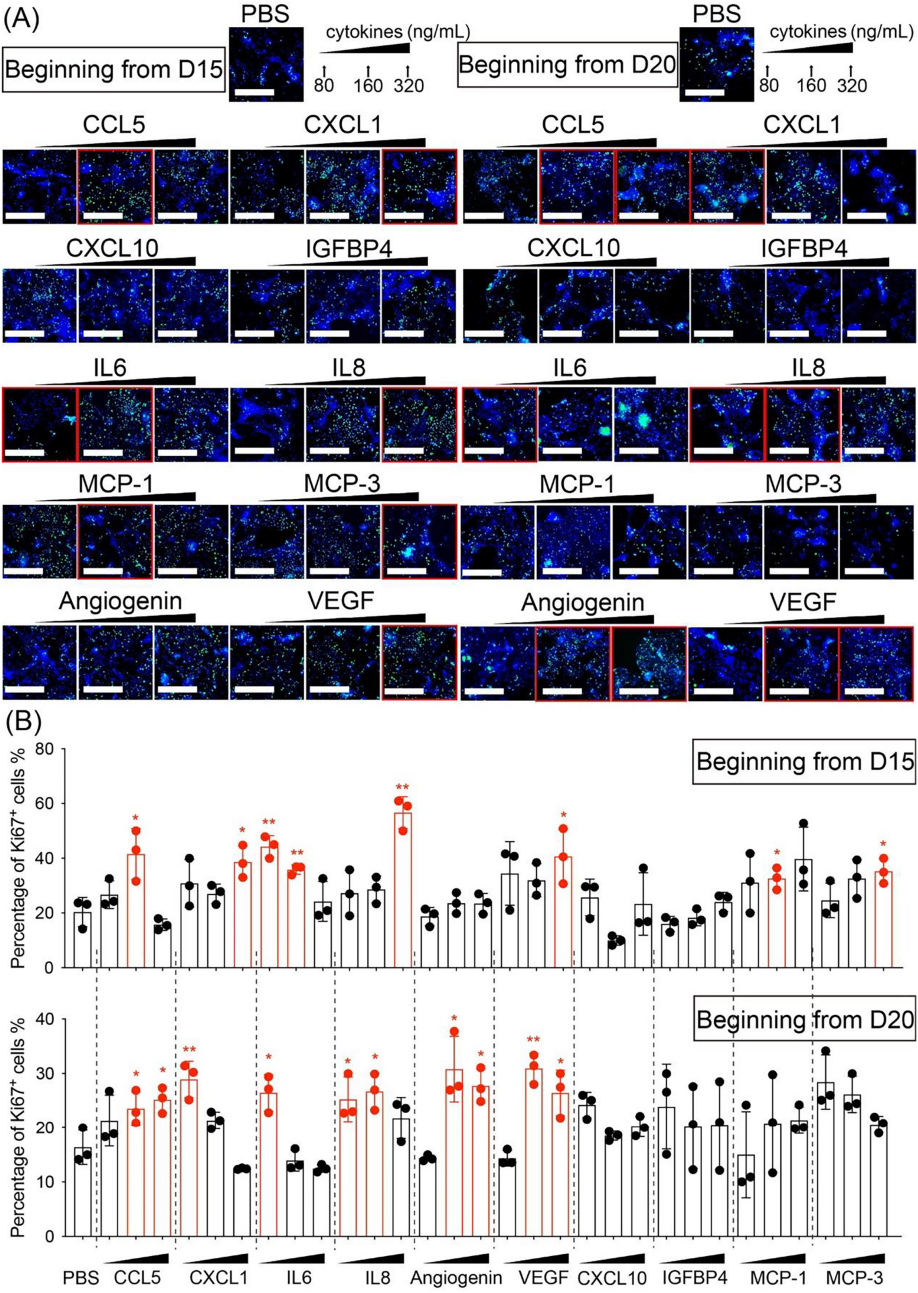
Immuno‐related cytokines enhanced the proliferation of mDA cells. (A) mDA cells on days 15 and 20 of differentiation were treated with different concentrations of cytokines, and the cell proliferation marker Ki67^+^ was detected after 4 days of incubation. Scale bar: 100 μm. (B) Quantification of Ki67^+^ in mDA cells treated with different concentrations of cytokines. Groups with significant differences compared to the PBS group at the same stage are marked in red. *p < 0.05, **p < 0.01. Student's *t*‐test was performed.

Further, we investigated the effects of the above cytokines at different concentrations on the proliferation of mDA cells. Different cytokines were supplemented at varying concentrations beginning from day 15 to day 20 of differentiation, and the medium with cytokines was refreshed every other day. As shown in Figure 6, treatment of mDA cells beginning from day 15 of differentiation with 160 ng/mL CCL5 (41.54% ± 9.30%, *p* = 0.0269), 320 ng/mL CXCL1 (38.65% ± 5.89%, *p* = 0.0164), 80 ng/mL IL6 (44.28% ± 3.96%, *p* = 0.0034), 160 ng/mL IL6 (35.84% ± 1.70%, *p* = 0.0088), 320 ng/mL IL8 (56.67% ± 5.84%, *p* = 0.0014), 320 ng/mL VEGF (40.71% ± 10.05%, *p* = 0.0364), 160 ng/mL MCP‐1 (32.62% ± 4.02%, *p* = 0.0339), and 320 ng/mL MCP‐3 (35.25% ± 4.59%, *p* = 0.0217) for 4 days resulted in a significant increase in the proliferation of mDA cells compared to the PBS‐treated group at the same stage (20.37% ± 5.35%). Furthermore, beginning from day 20 of differentiation, the addition of 160 ng/mL CCL5 (23.56% ± 3.07%, *p* = 0.0477), 320 ng/mL CCL5 (25.19% ± 2.37%, *p* = 0.0181), 80 ng/mL CXCL1 (28.93% ± 3.32%, *p* = 0.0091), 80 ng/mL IL6 (26.46% ± 3.36%, *p* = 0.0193), 80 ng/mL IL8 (25.25% ± 4.12%, *p* = 0.0416), 160 ng/mL IL8 (26.73% ± 3.33%, *p* = 0.0175), 160 ng/mL Angiogenin (30.79% ± 6.02%, *p* = 0.0214), 320 ng/mL Angiogenin (27.74% ± 3.12%, *p* = 0.0115), 160 ng/mL VEGF (30.95% ± 2.73%, *p* = 0.0038), and 320 ng/mL VEGF (26.44% ± 4.18%, *p* = 0.0293) for 4 days significantly increased cell proliferation. Overall, these results suggest that cytokines such as CCL5, CXCL1, IL6, IL8, VEGF, Angiogenin, and MCP‐3 may play a role in promoting the proliferation of mDA cells.

## 4 Discussion

Our study found that the proliferation of mDA cells was significantly enhanced when cocultured with medulloblastoma. These proliferating cells were predominantly early neural precursor cells, exhibiting a higher percentage of Ki67 and SOX1 both in vitro and in vivo, suggesting that abnormal proliferation was induced in the cocultured mDA cells. Although these abnormally proliferative cells did not meet the threshold for tumor formation in animal studies, the abnormal proliferation of cocultured mDA cells in vitro and in vivo grafts raises concerns about the potential risk for tumor formation. Further analysis revealed that the activation of the JAK/STAT3 pathway, cytokine stimulation (such as CCL5, CXCL1, IL6, IL8, VEGF, Angiogenin, and MCP‐3), and altered expression of metabolites (such as acetylcarnitine and carnitine) contribute to the enhanced proliferation of cocultured mDA cells.

We concluded that exposing mDA cells to the TME in vitro before transplantation could induce abnormal proliferation both in vitro (as indicated by cell density, proliferating markers, and colony formation, Figure 2) and in vivo grafts (proliferating markers, Figure 3). Our findings suggest that the TME can act as an inducer of abnormal proliferation in functional progenitors, thereby enhancing the sensitivity for detecting tumorigenicity. Compared to a normal microenvironment, the in vitro supplementation of the TME creates a more favorable environment for the proliferation of cells with tumorigenic potential. In the future, implementing an in vitro coculture model could enable the creation of an adjustable microenvironment for transplanted cells, significantly enhancing the detection sensitivity for potential risks associated with the transplanted cells.

Our findings demonstrated that cytokines, as components of the TME, contributed to the abnormal proliferation of cocultured mDA cells through hyperactivation of the JAK/STAT3 pathway, leading to the upregulation of the downstream transcription factor MYC. Prior studies have shown that some of the cytokines detected in our study can contribute to cell proliferation, tumor initiation, and progression [35]. For example, IL6 plays a crucial role in cell proliferation, differentiation, and the development of tumorigenesis through the JAK/STAT3 pathway [36, 37, 38]. Additionally, IL8 promotes angiogenesis and cell proliferation through CXCR1/CXCR2 receptors, playing a key regulatory role in the TME [39]. Furthermore, CXCL1 has been implicated in tumor formation [40], participating in the proliferation, differentiation, and neurogenesis of stem cells in the nervous system [41]. CCL5 and MCP‐3, cytokines of the CC family, are known to promote cell proliferation and hold significance in TME [42]. VEGF and angiogenin also play roles in angiogenesis and tumor formation [43, 44].

In addition to these cytokines, we observed elevated levels of acetylcarnitine and carnitine in cocultured mDA cells. Recent evidence suggests that acetylcarnitine and carnitine contribute to energy metabolism [32, 33], anti‐apoptotic pathways, and neuroprotection in the brain [45, 46, 47], indicating that acetylcarnitine/carnitine metabolism may induce a higher level of energy supply and contribute to the increased abnormal proliferation of cocultured mDA cells.

Some limitations in this study should be addressed in future research. First, the specific cell type or cell population and transmission mechanisms responsible for the abnormal proliferation of cocultured mDA cells need to be elucidated. This understanding will enable the identification of potential risk cells and the development of corresponding safety strategies in PD cell therapy. Second, the combinations of cytokines mentioned in this study, as well as other cytokines that are highly expressed in the degeneration of dopamine neurons or in PD patients (e.g., IL1α, IL2, IL1β, TNFα, TGF‐β, and IFNγ [48]), require further investigation to validate their potential safety in grafted mDA cells.

## 5 Conclusion

We concluded that abnormal proliferation was induced in cocultured mDA cells, involving the activation of the JAK/STAT3 signaling pathway and stimulation by specific cytokines and metabolites, posing a potential risk for tumor formation. Thus, we suggest that exposing functional progenitors derived from hPSCs to the TME in vitro before transplantation offers a more refined approach to enhancing the detection sensitivity for tumorigenicity tests.

## Ethics Statement

The study received ethical approval from the Institutional Animal Care and Use Committee of the Department of Laboratory Animal Science, Fudan University (Approval number: 202009007S).

## Conflicts of Interest

The authors declare no conflicts of interest.

## Supporting information


Data S1.

## Data Availability

The data that support the findings of this study are available from the corresponding author upon reasonable request. The raw data of RNA‐sequencing is available on the database of Sequence Read Archive (PRJNA1066752).
