# Supplementary material for: The Abnormal Proliferation of Midbrain Dopamine Cells From Human Pluripotent Stem Cells Is Induced by Exposure to the Tumor Microenvironment
Source: CNS Neurosci Ther. 2024 Nov 19;30(11):e70117. doi: 10.1111/cns.70117 (PMC11576488; doi:10.1111/cns.70117)
Supplement: Supplementary file 1 — Data S1. [file CNS-30-e70117-s001.docx]

Supporting Information

**The abnormal proliferation of midbrain dopamine cells from human pluripotent stem cells is induced by exposure to the tumor microenvironment**

Jun Xue^1,2^, Dongyan Wu^3,4^, Yuting Bao^1,2^, Yifan Wu^1,2^, Xin Zhang^1,2^, Liang Chen^1,2^

**
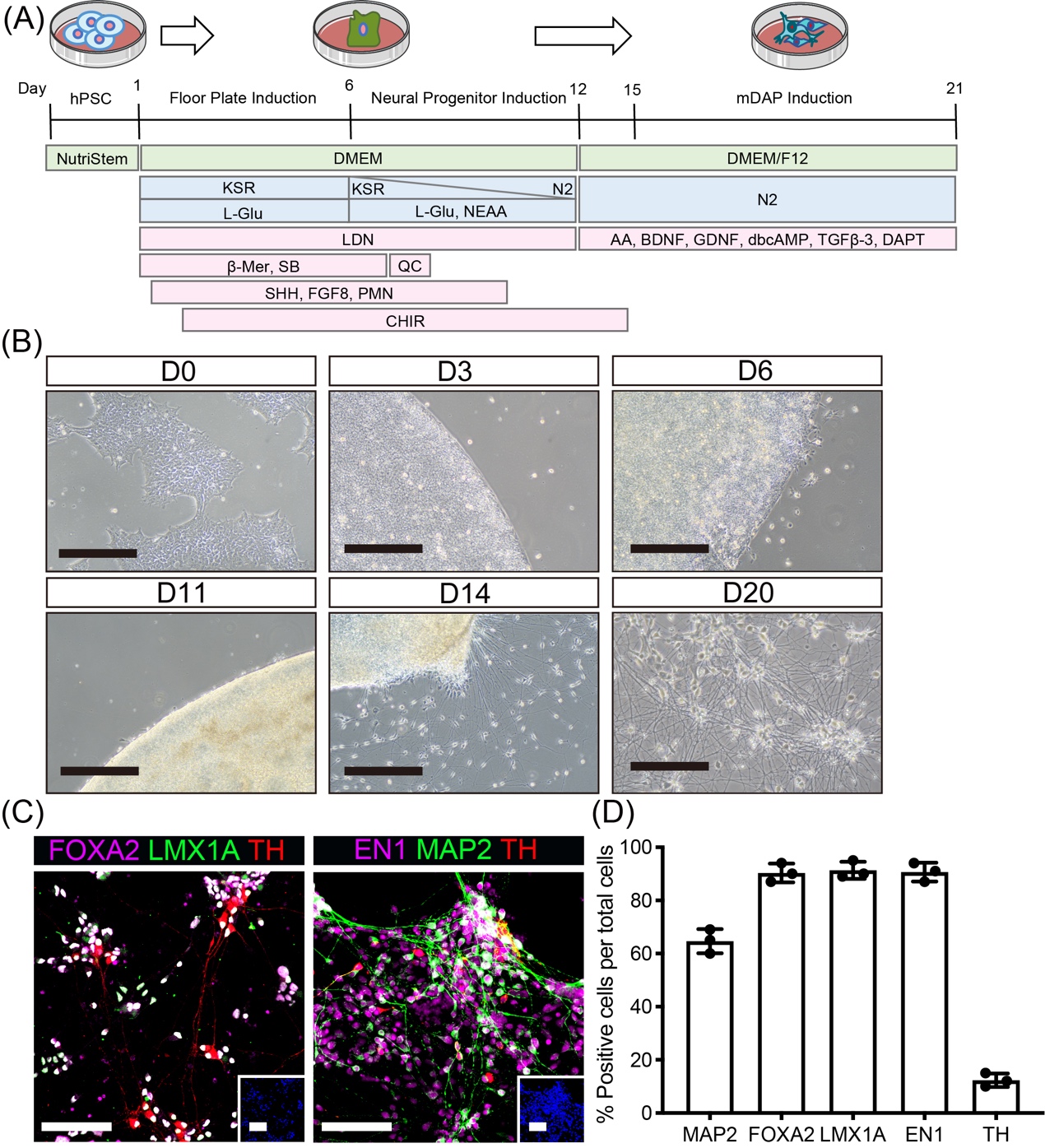
**

**FIGURE S1: Characterization of mDA cells derived from hPSCs.** (A) Schematic protocol of mDA cells differentiation. The detailed procedure and reagent concentration were indicated in the Methods section. (B) Bright-field images of differentiated mDA cells. Scale bar: 400 μm. (C) Representative immunofluorescence staining images of mature neurons (MAP2), mDAPs (FOXA2/LMX1A/EN1), and DANs (TH) at day 21 of differentiation. Scale bar: 80 μm. (D) Quantification of immunofluorescence marker populations in day 21 differentiated cells.

**
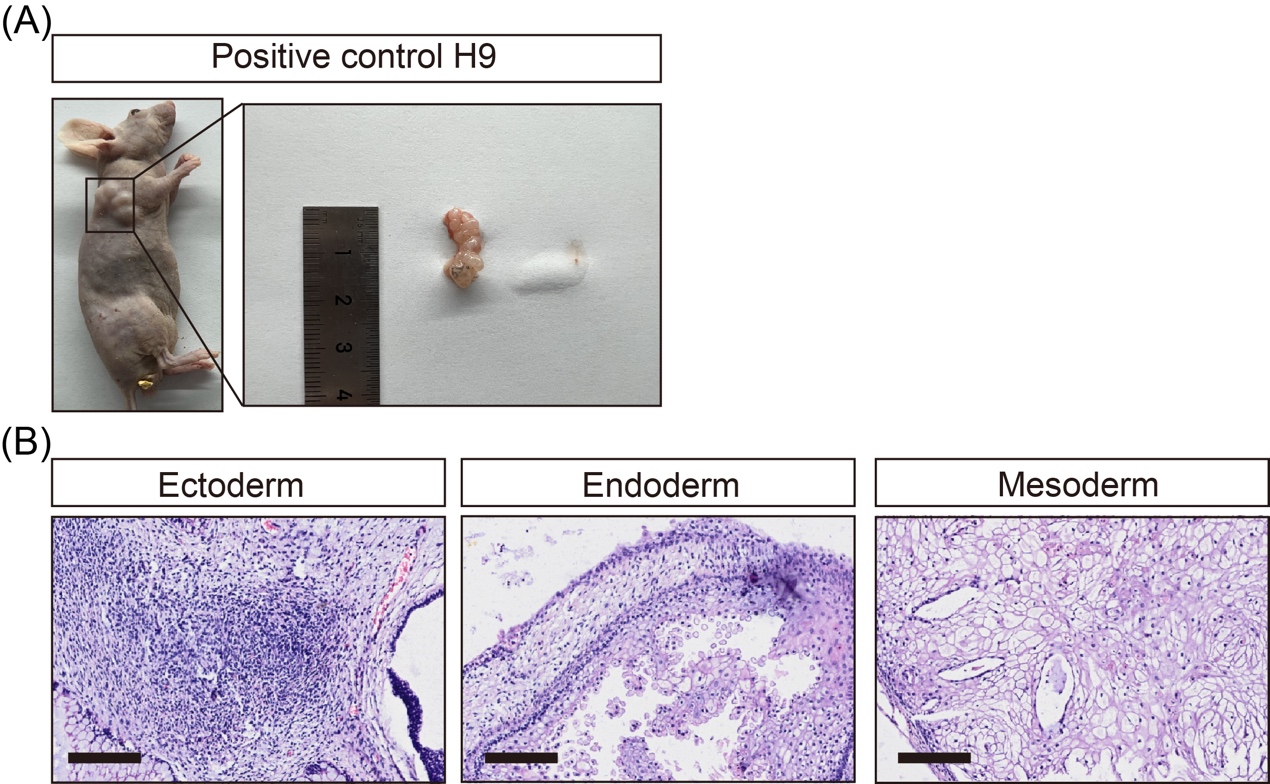
**

**FIGURE S2:** **Positive control H9 formed tumors in nude mice tumor formation assay.** (A) Representative images showing H9 injected subcutaneously into nude mice to form a tumor. (B) H&E staining showing the teratoma formation and the three germ layer tissues from the subcutaneous injection of H9. Scale bar: 200 μm.


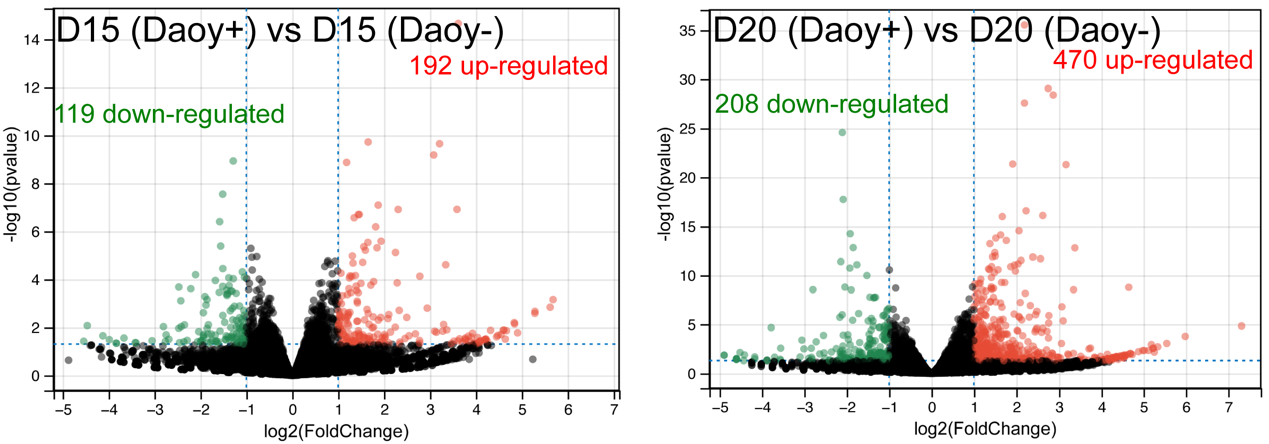


**FIGURE S3:** **Volcano plot for the gene libraries of the control and co-cultured groups.** Each dot represented an individual gene, red dots represented the up-regulated genes, green dots represented the down-regulated genes, and grey dots represented not differentially expressed genes.


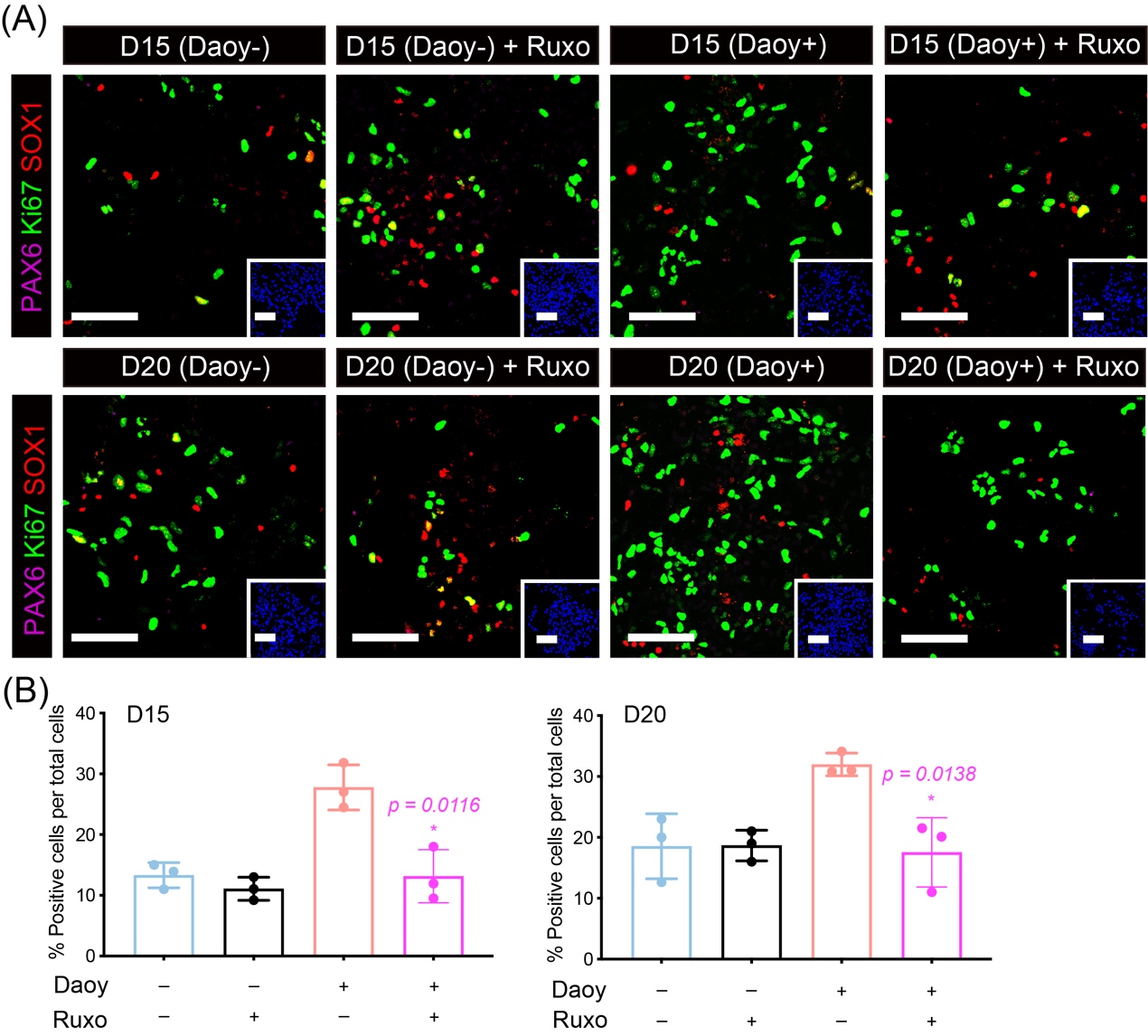


**FIGURE S4: Ruxolitinib could reverse the proliferation of co-cultured mDA cells.** (A) Immunofluorescence images showing Ki67, SOX1, and PAX6 in the control group, co-cultured group, control group treated with Ruxolitinib, and co-cultured group treated with Ruxolitinib. Scale bar: 80 μm. (B) Corresponding statistic percentage of Ki67^+^ populations in each group. * *p < 0.05*. Student’s t test was performed.

**Table S1. List of culture medium, chemicals, peptides, and recombinant proteins in this study**

| **Reagents** | **Company** | **Cat number** |
| --- | --- | --- |
| NutriStem medium | Biological Industries | 05-200-1A |
| Matrigel | Corning | 354277 |
| EDTA | Invitrogen | 15575-020 |
| MEM | Gibco | 11090081 |
| FBS | Gibco | 10099141C |
| NEAA | Gibco | 11140050 |
| GlutaMAX | Gibco | 5050061 |
| sodium pyruvate | Gibco | 11360070 |
| TrypLE | Gibco | 12605028 |
| accutase | Millipore | SCR005 |
| poly-L-ornithine | Sigma | P4957 |
| Fibronectin | Sigma | F0895 |
| Laminin | Sigma | L2020 |
| DMEM | Gibco | 11965092 |
| DMEM/F12 | Gibco | 11320033 |
| KSR | Gibco | 10828028 |
| L-Glu | Gibco | A2916801 |
| N2 supplement | Gibco | 17502048 |
| β-mercaptoethanoer | Thermo Fisher | 21985023 |
| Y27632 | TargetMol | T1725 |
| SB431542 | MedChemExpress | HY-10431 |
| LDN193189 | TargetMol | T6158 |
| SHH | Peprotech | 100-45-1 |
| FGF8 | Peprotech | 100-25 |
| PMN | MedChemExpress | HY-15108 |
| CHIR99021 | TargetMol | T2310 |
| AA | Sigma | A4403 |
| BDNF | Peprotech | 450-02 |
| GDNF | Peprotech | 450-10 |
| dbcAMP | Sigma | D0627 |
| TGF-β3 | Peprotech | 100-36E |
| DAPT | TargetMol | T6202 |
| IL6 | Sinobiological | 10395-HNAE |
| IL8 | Sinobiological | 10098-HNAE |
| IGFBP4 | Sinobiological | 10967-H08H |
| CXCL1 | Sinobiological | 10877-HNCE |
| CCL5 | Sinobiological | 10900-HNAE |
| MCP-1 | Sinobiological | 10134-H08Y |
| MCP-3 | Sinobiological | 11926-H07E |
| VEGF | MedchemExpress | HY-P7110A |
| Angiogenin | MedchemExpress | HY-P7503 |
| CXCL10 | MedchemExpress | HY-P7226 |
| Ruxolitinib | TargetMol | T1829 |

**Table S2. List of antibodies in this study**

| **Antibodies** | **Company** | **Cat number** | **Dilution** |
| --- | --- | --- | --- |
| anti-FOXA2 | Abnova | H00003170-M10 | 1:1000 |
| anti-LMX1A | Abcam | ab139726 | 1:1000 |
| anti-TH | Millipore | AB9702 | 1:2000 |
| anti-TH | Santa Cruz | sc-25269 | 1:2000 |
| anti-EN1 | DSHB | 4G11 | 1:1000 |
| anti-MAP2 | Cell signaling technology | 4542 | 1:1000 |
| anti-Histone H3 (phosphor S10) | Abcam | ab14955 | 1:500 |
| anti-Ki67 | Abcam | ab16667 | 1:2000 |
| anti-SOX1 | R&D | AF3369-SP | 1:500 |
| anti-PAX6 | Santa Cruz | sc-81649 | 1:500 |
| anti-MYC | Invitrogen | MA1-980 | 1:500 |
| anti-GABA | ImmunoStar | 20094 | 1:1000 |
| anti-GFAP | Dako | Z033401 | 1:1000 |
| anti-Iba-1 | Abcam | ab5076 | 1:1000 |
| anti-hNUMA | Abcam | ab97585 | 1:1000 |
| anti-STAT3 | Cell signaling technology | 9139 | 1:1000 |
| anti-phospho-STAT3 Try705 | Cell signaling technology | 9145 | 1:1000 |
| anti-GAPDH | Beyotime | AF0006 | 1:1000 |
| Hoechst 33258 | Abcam | ab228550 | 1:1000 |
| Cy3 anti-chicken | Jackson ImmunoResearch | 103-165-155 | 1:1000 |
| Alexa Flour 647 anti-mouse | Jackson ImmunoResearch | 715-606-151 | 1:1000 |
| Alexa Flour 488 anti-rabbit | Jackson ImmunoResearch | 711-545-152 | 1:1000 |
| Rhodamine anti-goat | Jackson ImmunoResearch | 705-296-147 | 1:1000 |
| anti-mouse, HRP conjugated | CWBIO | CW0102S | 1:3000 |
| anti-rabbit, HRP conjugated | CWBIO | CW0103S | 1:3000 |

**Table S3. List of ELISA kits in this study**

| ELISA Kit | Company | Cat number |
| --- | --- | --- |
| human IL6 ELISA Kit | Novus Biologicals | VAL102 |
| human IL8 ELISA Kit | Novus Biologicals | VAL103 |
| human CXCL1 ELISA Kit | Novus Biologicals | VAL139 |
| human VEGF ELISA Kit | Novus Biologicals | VAL106 |
| human Angiogenin ELISA Kit | Novus Biologicals | VAL158 |
| human MCP-1 ELISA Kit | Novus Biologicals | VAL134 |
| human CXCL10 ELISA Kit | Liankebio | EK168-96 |
| human CCL5 ELISA Kit | Liankebio | EK1129-96 |
| human MCP-3 ELISA Kit | Proteintech | KE00262 |
